# Efficacy of different organic and inorganic nutrient sources on the growth and yield of bitter gourd (*Momordica charantia* L.)

**DOI:** 10.1016/j.heliyon.2023.e22135

**Published:** 2023-11-10

**Authors:** Sudip Ghimire, Bidhya Poudel Chhetri, Jiban Shrestha

**Affiliations:** a Faculty of Agriculture (FOA), Agriculture and Forestry University, Rampur, Chitwan, Nepal; b National Plant Breeding and Genetics Research Centre, Khumaltar, Lalitpur, Nepal

**Keywords:** Bitter gourd, Chemical fertilizer, Farmyard, Fertilizers, Yield

## Abstract

The cultivation of bitter gourd (*Momordica charantia* L.) in Nepal faces significant challenges, resulting in diminished yields compared with other regions. The pivotal issue is optimizing fertilizer management practices to enhance bitter gourd growth and yield. In April 2022, a field experiment was conducted in Kapilvastu, Nepal to investigate the efficacy of organic and inorganic fertilizers on the growth and yield of bitter gourd. The study included five treatments: the recommended dose of nitrogen, phosphorus, and potassium (NPK) fertilizer (111.66:54.56:35.36 NPK kg ha^−1^, and 29.49 t ha^−1^ farmyard manure), 100 % poultry manure, 100 % farmyard manure, 50 % NPK + 50 % poultry manure, and 50 % NPK + 50 % farmyard manure. We hypothesized that a blend of 50 % organic and 50 % inorganic manure would significantly enhance bitter gourd performance. These treatments were evaluated in a randomized complete block design (RCBD) with four replications and analyzed using R-Studio. The results showed that 50 % NPK +50 % poultry manure increased growth parameters, such as plant height (380 cm), branch count plant^−1^ (22), node count plant^−1^ (34.75), female flower count (50.50), fruit length plant^−1^ (24.75 cm), and fruit diameter (6.53 cm), and decreased male flower count (132.25) and days to first male (33.75) and female (36.25) flowering. The application of 50 % NPK and 50 % poultry manure increased individual fruit weight (274.50 g) and yield-attributing traits, such as fruit count (17.25), fruit yield (55.56 t ha^−1^), net return (7944$), and benefit-cost ratio (3.14). A synergistic blend of 50 % NPK and 50 % poultry manure can serve as a viable and effective nutrient source for promoting growth and maximizing bitter gourd yield. Although these results are promising, further validation and extension of these positive findings are required on a larger scale in diverse ecological regions.

## Highlights

• Proper fertilizer management is crucial for optimal bitter gourd yield.
• The use of 50 % poultry manure +50 % NPK increased fruit length, diameter, weight, number and yield of bitter gourd.
• The integrated use of organic and inorganic fertilizers enhances the growth and yield potential of bitter gourd plants.

## Graphical abstract

**Figure:**
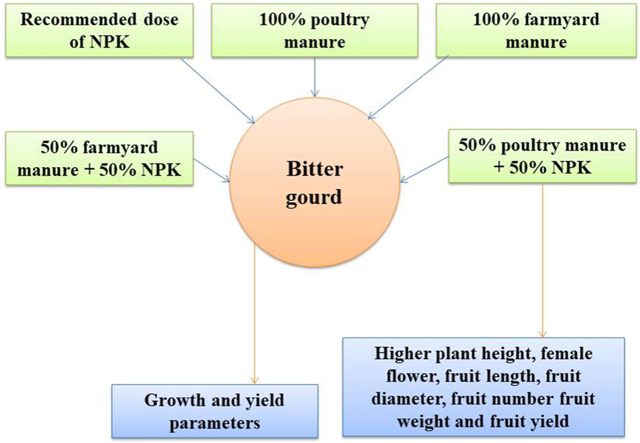

## 1 Introduction

The bitter gourd (*Momordica charantia* L*.*) is a balsam pear, bitter melon, bitter cucumber, and African cucumber [1], with a somatic chromosome number of 22 [[2], [3], [4]]. The bitter melon domestication center is most likely in eastern Asia, possibly in eastern India or southern China [[5], [6], [7]]. It is a rich source of iron, calcium, phosphorus, potassium, vitamin A, and vitamin C [8,9] and is high in fiber and low in calories [10,11]. Its juice consumption is also beneficial for diabetics, patients with high blood pressure, and patients with digestive issues owing to its powerful oxygen-free radical scavenging capability [[12], [13], [14], [15]] and improved insulin sensitivity [16,17]. Renowned for its versatility, bitter gourd finds its way into an array of culinary creations, particularly in Asian and African cuisine. This vegetable is commonly stir-fried, stuffed, or pickled, adding distinctive flavors and textures to dishes [18,19]. It has a bitter flavor that some people find difficult to get used to. Additionally, it is planted for decorative purposes and is widely used in traditional medicine [[18], [19], [20]]. In Nepal, bitter gourds are usually grown in kitchen gardens as summer vegetables. It is currently grown as a commercial crop in urban areas. Moreover, it can be grown in any type of soil with a good drainage system and soil pH range of 4.3–8.7 [21].

Fertilizer management has been identified as the main factor limiting agricultural productivity [22]. Owing to its remarkable responsiveness to fertilizers, bitter gourd has emerged as a crop that thrives when provided with an ample supply of essential plant nutrients. Therefore, it is crucial to cultivate bitter gourd plants in nutrient-rich environments to achieve optimal growth and productivity [23,24]. Nitrogen (N) promotes leaf and stem growth, and enhances the overall size and greenness of plants. It is a crucial component of proteins, chlorophyll, and other essential plant compounds [25,26]. Phosphorus (P) is essential for root development, flowering, fruiting, energy transfer, and storage, as well as the production of DNA and other vital molecules in plants [27]. Potassium (K) is a vital nutrient for plants and is involved in regulating water uptake, boosting disease resistance, enhancing photosynthesis, and promoting overall plant vigor by supporting various physiological processes [28]. Fertilizers exhibit variations in their chemical composition and quality, leading to diverse responses to soil application. These differential responses manifest in terms of soil chemical properties, crop production, and overall crop quality. Chemical fertilizer use is a key factor in agricultural food production [29,30]. Inorganic fertilizers may provide immediate benefits, but they are often not a sustainable long-term solution [31], as they can have negative environmental and health impacts, as well as diminishing returns over time, if not used carefully and in combination with other nutrient sources [29]. However, inorganic fertilizers are expensive, particularly for small-scale farmers. They also have severe impacts on human health, including chemical residues in food and water contamination, and they contribute significantly to environmental problems, such as excessive nutrient losses [32,33].

Organic manure, such as farmyard manure (FYM), has the potential to support cropping systems by promoting nutrient recycling and enhancing soil physical properties [[34], [35], [36]]. FYM provides plants with essential macro- and micronutrients, enriches the soil with organic matter, improves the relative C: N balance, and enhances the absorption of nutrients by plants, all of which collectively contribute to increased plant growth [37,38]. A noteworthy demonstration revealed that applying 20 tons of FYM ha^−1^ effectively alleviated the detrimental effects of drought stress by reducing evaporation, enhancing water retention, and increasing water-holding capacity, as it acts as mulch [37]. This beneficial outcome was attributed to the provision of crucial nutrients such as nitrogen, phosphorus, and potassium, leading to an enhanced quality of cumin essential oil [39]. The addition of poultry manure (PM) to soil improves both its physical characteristics and chemical composition [40]. Throughout the history of agriculture, PM has been utilized as a cost-effective and superior organic nutrient source for crops [41]. PM contains each of the 13 essential plant nutrients that plants need [42], can be used as a crop fertilizer to meet all or some of the demands [[43], [44], [45]], and has a residual effect on crops [28]. PM application increases the leaf count plant^−1^ and its surface area, which eventually results in a better photosynthetic rate and higher yield [44].

Compared to other cucurbits, bitter gourd exhibits a relatively low yield on a global scale [46], suggesting a possible research gap in enhancing production techniques. Increased nutrient use efficiency and reduced nutrient loss in agricultural systems, along with improving crop yield, are critical sustainability challenges in the present context [47]. Overreliance on chemical fertilizers has detrimental consequences on soil fertility and long-term natural productivity, as it diminishes the soil's inherent capacity to sustainably support plant growth [[48], [49], [50]], vegetable production has not had the long-term economic effect on farmers' livelihoods as expected. Furthermore, small-scale farmers may face challenges in providing the financial burden associated with purchasing chemical fertilizers. Bitter gourd growing with organic manure alone may result in delayed nutrient release [51], limiting root growth, whereas improper fertilizer usage during the transplanting and growth phases causes nutrient loss, environmental risks, and decreased production [29]. Information on precise guidelines for fertilizer and manure application is currently scarce [41]. In addition, the introduction of organic fertilizers to crop growers, coupled with guidance on minimizing chemical fertilizers using environmentally friendly alternatives, can facilitate the expansion of organic crops. A cost-effective integration of inorganic and organic fertilizers, that is, PM and FYM, can prove to be a sustainable solution from nutrient, soil status, fruit quality, and economic points of view [[52], [53], [54], [55]]. However, there is a dearth of research on cost-effective, environmentally sustainable, and specific nutrients blended with bitter gourd, indicating the need for further exploration and investigation in this area. This study delves into the effects of novel nutrient blends with local relevance, including combinations of 50 % NPK and 50 % organic manure, which offer distinctive insights into the synergistic potential of combining organic and inorganic sources. The objectives of this study were twofold: to evaluate the impact of organic and inorganic nutrient sources on bitter gourd growth and yield and to determine the optimal fertilizer dosage. The hypothesis posits that the use of a balanced organic-inorganic fertilizer blend will significantly improve bitter gourd growth and yield. The novelty of this study lies in the investigation of diverse fertilizer blends encompassing both organic and inorganic sources to optimize bitter gourd cultivation. Our approach aims to identify the most economically viable and environmentally sustainable options. Notably, this study introduces the innovative concept of a synergistic blend consisting of 50 % NPK and 50 % PM, demonstrating its promising impact on growth and yield. Furthermore, this study provides empirical evidence regarding the influence of fertilizer management on bitter gourds, offering quantitative data on growth parameters, yield-attributing traits, fruit quality, and the economics of production. The findings will provide both practical and theoretical value to the field, offering valuable recommendations to farmers to adopt sustainable and cost-effective practices that increase yields while reducing nutrient loss.

## 2 Materials and methods

### 2.1 Experimental site and cropping history

The research was conducted in an open area in Ramapur-6, Kapilvastu district, during the summer season of April 2022. The precise geographical coordinates of the site are 7°17′84″ N latitude, 84°72′13″ E longitude, and 197 m above the mean sea level. This area experiences an average annual rainfall of approximately 1850 mm, and the temperature is typically subtropical, ranging from 25 °C to 35 °C. The selected land had previously been planted with other vegetable crops such as tomatoes and brinjals. Prior to this, the land was radishes planted. This earlier culture might have influenced the results of the present experiment.

### 2.2 Soil and manure properties

The soil type at the experimental site was a sandy loam. Soil samples were extracted from the experimental plots to a depth of 0–30 cm. These samples were subsequently crushed and passed through a 2 mm mesh sieve prior to nutritional testing. The soil testing laboratory at Bhumahi, Nawalparasi, conducted physical and chemical analyses of the soil. The nutrient analyses of PM and FYM used during the experimental trial were conducted at the same laboratory and are shown in Table 1.

**Table 1:** Physio-chemical properties of the soil, nutrient analysis report of PM and FYM used.

S·N	Parameters	Test/method	Observed values
Physio-chemical properties of the soil			
1	PH	Electrometric	8.0
2	Total N (%)	Kjeldhal	0.063
3	Available P (kg ha^−1^)	Modified Olsen's bicarbonate	180.60
4	Available K (kg ha^−1^)	Flame-Photometer	134.40
Nutrient analysis report of PM used			
1	PH	Electrometric	7.2
2	Total N (%)	Kjeldhal UV visible	0.80
3	Total P (P_2_O_5_) (%)	Spectrophotometer	2.56
4	Total K (K_2_O) (%)	Flame-Photometer	2.70
Nutrient analysis report of FYM used			
1	PH	Electrometric	8.7
2	Total N (%)	Kjeldhal UV visible	0.38
3	Total P (P_2_O_5_) (%)	Spectrophotometer	1.13
4	Total K (K_2_O) (%)	Flame-Photometer	2.40

### 2.3 Experimental details

The experiment involved five distinct dosages of both organic and inorganic fertilizers, serving as the treatments (T1 = recommended dose of NPK fertilizer (111.66:54.56:35.36 NPK kg ha^−1^ and 29.49 t ha^−1^ FYM), T_2_ = 100 % poultry manure, T_3_ = 100 % farmyard manure, T_4_ = 50 % NPK + 50 % poultry manure, and T_5_ = 50 % NPK + 50 % farmyard manure), was evaluated in a Randomized Complete Block Design (RCBD) with four replications. The seeds of the Palee variety of bitter gourd (East-West Seed International, India), urea, DAP, MoP, poultry manure, and farmyard manure were collected from New Dikshya Agrovet, Pipara, Kapilvastu, Nepal. The FYM used in this experiment was cattle FYM. It was matured after 5 months of decomposition. The FYM consisted of 0.38 % total nitrogen, 1.13 % total phosphorous, and 2.40 % total potassium (Table 1). The PM consisted 0.80 % total nitrogen, 2.56 % total phosphorus, and 2.70 % total potassium.

In the experimental setup, 20 plots were utilized, each with a gross plot size of 4 m × 4 m. The distance between rows within each plot was maintained at 1 m, whereas the spacing between individual plants along the rows was set at 1 m. Each plot consisted of four rows contributing to the overall net area of the experimental field, which covered 425.5 square meters. For data collection and observation, four plants were selected from each plot. To ensure adequate separation and minimize potential interference between the experimental units, the distance between adjacent plots was maintained at 0.5 m. Similarly, a separation of 0.5 m was established between replications, thereby providing a systematic and controlled layout for conducting the research.

### 2.4 Field preparation and seed sowing

The field was prepared by harrowing it twice after plowing with a tractor-drawn disc plow. After a 24-h soaking period, two seeds were planted in the soil at a depth of 15 cm. One seedling of low quality was removed after emergence, leaving healthy seedlings to continue to develop.

### 2.5 Application of manure and fertilizers

FYM and PM were administered during sowing from the estimated values (T_1_ = 314 g urea, 188.70 g DAP, 94.35 g MoP, 47.17 kg FYM, T_2_ = 30 kg PM, T_3_ = 63.15 kg FYM, T_4_ = 15 kg PM, 264 g urea, 188.70 g DAP, 94.35 g MoP, and T_5_ = 31.58 kg FYM, 264 g urea, 188.70 g DAP, 94.35 g MoP), whereas urea fertilizer was administered in divided or split doses throughout the growth period. Based on AIATC [56], the weight of inorganic fertilizer (urea, DAP, and MoP) was calculated based on the required NPK dosage for bitter gourds. The weight of organic fertilizers such as PM and FYM was determined using laboratory test findings. The N% content of the manure was used as a guideline for application in the field.

### 2.6 Cultural operations for bitter gourd

#### 2.6.1 Gap filling and intercultural operation

The diseased and frail plants were removed. Sound seedlings were allowed to continue growing in the field, and the appropriate procedures for weeding, watering, and staking were followed. During the dry season, the experimental field received continuous irrigation at intervals of 6–7 days beginning 15 days after sowing (DAS) following germination.

#### 2.6.2 Disease and pest management

Appropriate management practices were used depending on the severity and infestation of the illness and insects. Fruit flies were managed by spraying Malathion 50 EC in 1.5–2 ml L^−1^ water. The neighboring field was equipped with pheromone traps and bait to manage insects.

#### 2.6.3 Harvesting

Once the fruit reached its proper form and size, it was manually harvested. Before the seeds fully grew and hardened, and the skin became a consistent shade of green, bitter gourds were picked when they were almost full size. The fresh weight of each subsequent harvest was recorded individually on alternate days.

### 2.7 Growth parameters

#### 2.7.1 Plant height, branch count plant^−1^, node count plant^−1^

The heights of the four tagged plants from each plot were measured using a meter scale and reported in centimeters at 30 DAS. Further data was gathered at 15-day intervals to determine the mean height of the plant throughout its development period up to 75 DAS. The branch count on the tagged plant was recorded after 30 DAS, and this data collection was repeated at 15-day intervals. Similarly, the node count from the main stem was conducted once the plant completed blooming at 30 DAS, and subsequent data were collected every 15 days thereafter. This systematic data collection allowed the growth and development of plant branches and nodes to be tracked over time.

#### 2.7.2 Days to first male and female flowering, male flower count, and female flower count

The number of days to the first male and female blooming was observed and recorded for each treatment, and the mean values were computed. Following the first bloom, male and female flowers were counted every 15 days throughout the culture period. This constant monitoring allows for the tracking of blooming patterns and the evaluation of the plants' total flower yield.

### 2.8 Yield and yield-attributing parameters

#### 2.8.1 Fruit count plant^−1^, fruit length (cm), diameter (cm), and weight (g)

At the time of harvesting, the fruit count plant^−1^ was recorded in the dataset, specifically for the selected plants. In particular, the tagged plant provided measurements of the fruit's length, diameter, and weight when it reached the market-ready stage. Based on these measurements, the mean data were generated, allowing for an assessment of the average fruit characteristics of the selected plants.

#### 2.8.2 Fruit yield (t ha^−1^)

The yield calculation involved weighing fruits collected from multiple harvests. The cumulative weight of all fruits harvested from a single plant throughout the season was used to determine the yield plant^−1^. Once the entire harvest was complete, the overall yield in tons per hectare (t ha^−1^) was recorded.

### 2.9 Statistical analysis

The data collected from the experimental plots of various parameters were subjected to statistical analysis. The data were tabulated using Microsoft Excel 2010 (version 14.0.4734.1000), and analysis was conducted using R Studio (version R-3.6.3). Duncan's Multiple Range Test (DMRT) was used to detect significant differences between mean values at a significance level of 5 %. DMRT was utilized to distinguish means that exhibited significant variations, while an analysis of variance (ANOVA) was conducted to determine the overall significance of the data based on the structure of the ANOVA table.

## 3 Results and discussion

### 3.1 Growth parameters

#### 3.1.1 Plant height

Table 2 displays plant height data at 30, 45, 60, and 75 DAS for the various treatments. The simultaneous application of organic and inorganic nutrient sources significantly influenced plant height, surpassing the effects of the individual applications. Average plant height varied between 83.10 cm at 30 DAS and 347.65 cm at 75 DAS. The treatment with 50 % NPK +50 % poultry manure resulted in the highest plant height (160 cm), whereas the treatment with 100 % farmyard manure had the lowest height (131 cm) at 45 DAS. At 75 DAS, the 50 % NPK +50 % poultry manure treatment showed the highest height (380 cm), followed by 50 % NPK + 50 % farmyard manure (356.75 cm), whereas poultry manure (342.75 cm), the recommended dose of NPK (335.25 cm), and farmyard manure alone (323.50 cm) had lower heights.

**Table 2:** Plant height influenced by different organic and inorganic fertilizers.

Nutrient sources	Plant height (cm)			
	30 DAS	45 DAS	60 DAS	75 DAS
Recommended dose of NPK	74.50^b^	132.75^b^	229.25^c^	335.25^bc^
100 % poultry manure	88.00^a^	151.75^a^	240.50^bc^	342.75^bc^
100 % farmyard manure	71.50^b^	131.00^b^	228.75^c^	323.50^c^
50 % NPK + 50 % poultry manure	90.75^a^	160.00^a^	272.50^a^	380.00^a^
50 % NPK + 50 % farmyard manure	90.75^a^	155.25^a^	252.25^b^	356.75^ab^
Grand Mean	83.10	146.15	244.65	347.65
SEm (±)	1.81	2.75	5.05	9.50
LSD (0.05)	5.59	8.48	15.56	29.29
F-test	***	***	***	*
CV%	4.36	3.76	4.12	5.47

Data in columns with the same letters in DMRT are not significantly different (p = 0.05); SEm (±) = standard error of the mean; CV = coefficient of variation; LSD = least significant difference; * = significant at p < 0.05; *** = significant at p < 0.001.

Evaluation of the effect of organic manure on plant height and vegetative development can serve as an indicator of its contribution to soil fertility, particularly in soils with low carbon content [57]. Limited nitrogen (N) contained in sole FYM could lead to growth retardation in plants due to reduced accessibility of photosynthetic pigments, hindered carbon fixation, and compromised water relations [26], due to which sole FYM application resulted in the lowest height. The combination of NPK fertilizers, which contain essential nutrients such as N, P, and K, along with PM, which is rich in organic matter and various nutrients, including nitrogen, phosphorus, potassium, and micronutrients, provides a balanced and comprehensive nutrient supply from source to growing points [[42], [43], [44], [45]]. Significant vine growth was observed when divided urea doses were administered [57]. Moreover, the nitrogen present in PM is readily accessible to plants because approximately 30 % of the N in PM is in the form of nitrate or ammonium [58,59]. The availability of N in easily absorbable forms likely contributed to the effectiveness of poultry manure as a nutrient source for plants. This balanced nutrient combination, linked to strong photosynthetic activity, root development, and increased carbohydrate translocation from the source to the growing point, likely played a significant role in promoting optimal plant growth and development [57]. PM generally has a higher mineral nutrient content, including nitrogen, calcium, phosphorus, zinc, and magnesium, than FYM [60]. Subedi et al. [61], Basnet et al. [58], and Devkota et al. [62] noted that the synergistic application of chemical fertilizer and poultry manure (PM) resulted in the highest plant heights observed in radish and broadleaf mustard, respectively. The higher mineral nutrient content in PM makes it a valuable organic fertilizer for plants, providing a rich source of essential nutrients for growth and development. Moreover, they stimulate cell division and elongation, leading to increased growth and mineral content when incorporated into inorganic fertilizers [37,63].

#### 3.1.2 Branch count plant^−1^

The branch count plant^−1^ was significantly influenced by the application of different fertilizers throughout the observation period. The average branch count plant^−1^ ranged from 2.20 at 30 DAS to 18.90 at 75 DAS. The treatment with 50 % NPK +50 % poultry manures consistently had the highest branch count at 30, 45, 60, and 75 DAS, which was statistically similar to the treatment with 50 % NPK +50 % farmyard manure. Specifically, at 75 DAS, the 50 % NPK +50 % poultry manure treatment had the highest branch count (22), followed by 50 % NPK + 50 % farmyard manure (20), while poultry manure (17.75), recommended dose of NPK (17.50), and farmyard manure (17.25) had the lowest branch count, as presented in Table 3.

**Table 3:** Branch count plant^−1^ influenced by different organic and inorganic fertilizers.

Nutrient sources	Branch count plant^−1^			
	30 DAS	45 DAS	60 DAS	75 DAS
Recommended dose of NPK	2.25^ab^	6.00^ab^	13.25^ab^	17.50^b^
100 % poultry manure	1.50^b^	5.75^b^	11.00^bc^	17.75^b^
100 % farmyard manure	1.75^b^	4.75^b^	10.25^c^	17.25^b^
50 % NPK +50 % poultry manure	3.00^a^	7.5^a^	14.00^a^	22.00^a^
50 % NPK +50 % farmyard manure	2.50^ab^	5.25^b^	11.00^bc^	20.00^ab^
Grand Mean	2.20	5.85	11.9	18.90
SEm (±)	0.35	0.53	0.9	1.37
LSD (0.05)	1.07	1.70	2.77	4.22
F-test	*	*	*	*
CV%	31.81	18.91	15.12	14.50

Data in columns with the same letters in DMRT are not significantly different (p = 0.05); SEm (±) = standard error of the mean; CV = coefficient of variation; LSD = least significant difference; * = significant at p < 0.05.

NPK fertilizers contain essential macronutrients such as nitrogen, phosphorus, and potassium, whereas poultry manure enriches the soil with organic matter, micronutrients, and beneficial microorganisms. In the early stages of growth, when a plant has an immature root system and a restricted number of branches and leaves, the sole application of urea can lead to increased nutrient loss and insufficient uptake by the plant. Nevertheless, the incorporation of PM enables the gradual release of nutrients, affording the plant ample time to absorb and utilize these nutrients efficiently, resulting in a higher branch plant^−1^ [57]. Plants can easily absorb nitrogen present in PM, in contrast to FYM [58,59]. Additionally, poultry manure contains growth-promoting substances such as auxins, cytokinin, and gibberellins [[64], [65], [66]], which can stimulate cell division, elongation, and branching in plants [67]. Plant growth regulators are instrumental in facilitating the growth of lateral shoots, thereby augmenting the branch and node count, and the increase in vegetative metrics, such as primary branch count, may be attributable to the involvement of nitrogen in encouraging vegetative development, improving cell division and elongation, and increasing chlorophyll production [68]. The balanced nutrient availability to the plants due to the integration of NPK and PM, which produced a good soil environment, might be the cause of the increase in the branch count because nitrogen is readily accessible to plants when provided in an equal mixture of fertilizer sources [57]. Previous studies have underscored the significant influence of organic fertilizers on plant development and growth, nutrient absorption, and photosynthetic efficiency. These effects can be ascribed to the increased activity of enzymes such as alkaline phosphatase and acid phosphatase [69]. Organic fertilizers can enhance soil chemical properties including cation exchange capacity (CEC), pH, and nutrient availability [37]. The integration of such fertilizers with inorganic fertilizers enhances soil fertility and overall fertility by augmenting organic matter content and improving nutrient availability in the soil [37]. The integration of organic fertilizers, such as PM and FYM, influences physiological processes by enhancing enzyme activity and facilitating the transfer of photosynthetic products. This mixture enhances the soil organic content and leads to improved yield components such as an increased number of auxiliary branches [37].

#### 3.1.3 Node count plant^−1^

The statistical analysis revealed a noteworthy influence of the fertilizer on the node count at 30 and 45 DAS, as illustrated in Table 4. On average, the node count plant-1 ranged from 13.15 at 30 DAS to 31.55 at 75 DAS. At 30 DAS, the treatment with 50 % NPK +50 % poultry manure had the highest node count plant-1 (15), which was significantly different from the other treatments. The lowest node count was observed after treatment with FYM (11.25). At 75 DAS, the treatment with 50 % NPK +50 % poultry manure had the highest node count plant-1 (34.75), which was statistically comparable to the treatment with 50 % NPK + farmyard manure (33.75), while the treatment with farmyard manure alone had the lowest node count plant-1 (27.75)*.*

**Table 4:** Node count plant^***−1***^ influenced by different organic and inorganic fertilizers.

Nutrient sources	Node count plant^*−*^*^1^*			
	30 DAS	45 DAS	60 DAS	75 DAS
Recommended dose of NPK	12.25	18.25	22.75^b^	28.00^b^
100 % poultry manure	13.00	18.50	28.00^a^	33.50^a^
100 % farmyard manure	11.25	18.75	22.75^b^	27.75^b^
50 % NPK +50 % poultry manure	15.00	22.5	30.00^a^	34.75^a^
50 % NPK +50 % farmyard manure	14.25	21.75	28.00^a^	33.75^a^
Grand Mean	13.15	19.95	26.30	31.55
SEm (±)	0.96	1.93	0.88	0.95
LSD (0.05)	2.96	5.96	2.73	2.93
F-test	NS	NS	***	***
CV%	14.62	19.39	6.73	6.03

Data in columns with the same letters in DMRT are not significantly different (p = 0.05); SEm (±) = standard error of the mean; CV = coefficient of variation; LSD = Least Significant Difference; *** = significant at p < 0.001; NS = Non-significant.

The synergistic utilization of organic and chemical fertilizers has been found to augment the activity of acid and alkaline phosphatase enzymes in the vicinity of plant roots [37]. This enzymatic activity not only leads to an increase in soil phosphorus content, but also facilitates the uptake of N, zinc (Zn), copper (Cu), and iron (Fe) by plants [70], which ultimately contributes to an increase in node count. The absorption of nutrients, notably nitrogen, promotes cell division and elongation, resulting in rapid growth, and is freely available to plants in an equal mix of organic manure (PM) sources [58]. This synergistic effect of fertilizers promotes improved nutrient availability and uptake, thereby supporting plant growth and development [57]. These improvements promote increased root development and overall vegetative growth, including node count plant^−1^. The obtained results provide compelling evidence that the synergistic application of organic and chemical fertilizers is a successful strategy for modifying soil fertility and improving nutrient uptake [57]. The implementation of this approach has the potential to enhance the quantitative and qualitative characteristics of bitter gourds.

#### 3.1.4 Days to first male and female flowering, male and female flower counts

The application of different fertilizers had a significant effect on the timing of the first male and female flowers (Table 5). On average, the first male flowering occurred at approximately 36.5 days, while the first female flowering occurred at approximately 40.5 days. Treatments with 50 % NPK +50 % poultry manure and 50 % NPK +50 % farmyard manure resulted in the shortest duration for the first male flowering (33.75 days), which was statistically similar. The treatment with 50 % NPK +50 % poultry manure had the shortest duration for first female flowering (36.25 days), which was statistically similar to the treatment with 50 % NPK + 50 % farmyard manure (37.50 days). The longest duration for the first female flowering was observed with the sole application of farmyard manure (45 days).

**Table 5:** Days to first male and female flowering, and male and female flower count influenced by different organic and inorganic fertilizers.

Nutrient sources	Days to first male flowering	Days to first female flowering	Male flower count	Female flower count
Recommended dose of NPK	38.75^a^	43.00^ab^	147.75^ab^	42.00^c^
100 % poultry manure	35.25^b^	40.75^bc^	143.25^abc^	44.25^bc^
100 % farmyard manure	41.00^a^	45.00^a^	153.25^a^	41.75^c^
50 % NPK +50 % poultry manure	33.75^b^	36.25^d^	132.25^c^	50.50^a^
50 % NPK +50 % farmyard manure	33.75^b^	37.50^cd^	136.00^bc^	45.75^b^
Grand Mean	36.5	40.5	142.5	44.85
SEm (±)	1.08	1.17	4.09	0.93
LSD (0.05)	3.32	3.62	12.61	2.87
F-test	**	***	*	***
CV%	5.92	5.81	5.74	4.16

Data in columns with the same letters in DMRT are not significantly different (p = 0.05); SEm (±) = standard error of the mean; CV = coefficient of variation; LSD = least significant difference; * = significant at p < 0.05; ** = significant at p < 0.01; *** = significant at p < 0.001.

The main problem with bitter gourds is the male-to-female flower ratio [71]. Statistical analysis revealed significant differences in male and female flower counts among all treatments. Farmyard manure exhibited the highest male flower count (153.25), followed by NPK (147.75) and poultry manure (143.25). The treatment with 50 % NPK +50 % poultry manure had the lowest number of male flowers (132.25), which was statistically similar to the treatment with 50 % NPK + 50 % farmyard manure (136). In terms of female flowers, the treatment with 50 % NPK +50 % poultry manure produced the highest number of flowers (50.50), followed by 50 % NPK + 50 % farmyard manure (45.75), poultry manure (44.25), and the recommended dose of NPK (42), while farmyard manure had the lowest female flower count (41.75)*.*

Flowering is dependent on the availability of nutrients required for the development of reproductive tissues and fruits, making nutrient availability crucially interconnected with this reproductive process [72]. There is a tendency for a reduction in overall reactive nitrogen losses when inorganic nitrogen fertilizers are partially replaced with organic manure, as evidenced by studies conducted [53,73]. The optimal uptake of N not only promotes nutrient availability but also imposes physiological and developmental constraints. These constraints can arise from ion-specific toxicity or indirect limitations on carbon fixation and energy availability [26], which ultimately accelerates days to flowering and fruiting. The reduced male flower count and higher female flower count (sex ratio) may be due to balanced nutrition from organic and inorganic manure sources [74]. The organic nature of poultry manure, along with an adequate nitrogen supply, introduces beneficial microorganisms and organic compounds that can influence hormonal regulation in plants [75], which can positively influence flower development and reproductive processes. Moreover, mixing organic manure, such as PM, increases soil water retention [28], which, in turn, is responsible for a higher female flower count [76,77]. The incorporation of PM affects physiological processes by increasing enzyme activity and facilitating the translocation of photosynthetic products, leading to earlier flowering and a higher female flower count. Conversely, in other treatments where nutrient loss occurred and soil quality for root development was inferior to that of the treatment involving integrated fertilizer application, the flowering process may experience delays, consequently leading to an extended duration for flowering and fruiting and a higher male flower count. Utilizing PM, FYM, and NPK not only improved soil physical properties but also fostered the development of a robust root system, resulting in enhanced nutrient and water absorption. This integrated nutrient management ultimately contributes to accelerated plant growth and earlier onset of flowering and fruiting [57].

### 3.2 Yield parameters

#### 3.2.1 Fruit length, diameter, weight, and fruit number

Treatment with 50 % NPK +50 % poultry manure resulted in a maximum fruit length of 25 cm, which was statistically similar to that of the treatment with 50 % NPK +50 % farmyard manure (24.75 cm). The poultry manure had a slightly shorter fruit length (20.75 cm). The farmyard manure treatment had a minimum fruit length of 17.75 cm. In terms of fruit diameter, the highest values were observed with 50 % NPK +50 % poultry manure (6.67 cm), followed by 50 % NPK + 50 % farmyard manure (6.53 cm) and poultry manure (6.25 cm). For fruit weight, the treatments with 50 % NPK +50 % poultry manure and 50 % NPK +50 % farmyard manure showed significant differences compared to the other treatments. The heaviest fruit weight was recorded with 50 % NPK +50 % poultry manure (274.50 g), followed by 50 % NPK + 50 % farmyard manure (274.00 g) and poultry manure (262.25 g). The farmyard manure treatment had the lowest fruit weight (245.75 g). The highest number of fruits was observed with 50 % NPK +50 % poultry manure and 50 % NPK +50 % farmyard manure (17.25), followed by poultry manure (15.75) (Table 6).

**Table 6:** Fruit length, diameter, weight and count plant^−1^ influenced by different organic and inorganic nutrient sources.

Nutrient sources	Fruit length (cm)	Fruit diameter (cm)	Fruit weight (g)	Fruit count plant^−1^
Recommended dose of NPK	19.50^bc^	5.83^b^	253.75^b^	13.75^b^
100 % poultry manure	20.75^b^	6.25^ab^	262.25^ab^	15.75^ab^
100 % farmyard manure	17.75^c^	5.83^b^	245.75^b^	13.25^b^
50 % NPK + 50 % poultry manure	25.00^a^	6.67^a^	274.50^a^	17.25^a^
50 % NPK + 50 % farmyard manure	24.75^a^	6.53^a^	247.00^a^	17.25^a^
Grand Mean	21.55	6.22	262.05	15.45
SEm (±)	0.92	0.22	5.45	0.94
LSD (0.05)	2.83	0.68	16.81	2.92
F-test	***	*	*	*
CV%	8.54	7.18	4.16	12.28

Data in columns with the same letters in DMRT are not significantly different (p = 0.05); SEm (±) = standard error of the mean; CV = coefficient of variation; LSD = least significant difference; * = significant at p < 0.05; *** = significant at p < 0.001.

Our findings are in contrast to those of Tao et al. [55], who reported that the addition of chemical fertilizer to PM application treatments did not have a positive effect on the number of cherry tomatoes, which could be attributed to methodological differences (chemical fertilizers used, the timing and frequency of their application, or the specific cultivars), variability in plant response, environmental factors, or chance variation. Integrating organic manures containing N, P, and K has the potential to accelerate chlorophyll and amino acid production and enhance the translocation of photosynthetic products from leaves to fruits [[42], [43], [44], [45]]. This, in turn, can lead to an increase in length, diameter, weight, and number of fruits. Furthermore, poultry manure releases nutrients slowly over time, ensuring a sustained nutrient supply throughout the plant growth cycle. The equal mixture of different fertilizers resulted in higher yield-attributing characteristics, which might be because organic fertilizers provided sufficient nutrients and were delivered slowly into the plants when mixed with high-nutrient-containing inorganic fertilizer, which increased the physio-morphological attributes of the fruit [78]. This decreased nutrient loss and leaching enhances the effectiveness of nutrient use [54,78]. When compared to sole organic and inorganic fertilizers, the use of integrated nutrients (50 % NPK + 50 % poultry manure) in bitter gourd cultivation clearly improved the fruit quality, as coupling PM with NPK contributed to higher availability of more than 13 important micro-and macronutrients [42]. The observed differences in fruit measurements among the treatments can be attributed to enhanced root development and effective nutrient uptake during the early growth stages, as well as increased nitrogen availability during the reproductive phase or fruiting, achieved through split-dose urea application [57]. Utilizing mixed fertilizers enhances nutrient accessibility for bitter gourd crops compared to using individual fertilizers [57]. Conversely, FYM alone exhibited the smallest fruit length, diameter, weight and number likely because of insufficient nitrogen availability needed for optimal fruit growth. The incorporation of organic manure with NPK helped mitigate nutrient loss and leaching, thereby enhancing nutrient utilization, and ultimately resulting in larger fruit measurements [57].

#### 3.2.2 Fruit yield

The statistical analysis revealed significant differences in fruit output among the treatments. The treatment with 50 % NPK +50 % poultry manure resulted in the highest fruit yield, reaching 55.56 t ha^−1^ (Table 7). This was followed by the treatment with 50 % NPK +50 % farmyard manure, which yielded 51.55 t ha^−1^ of fruit. The poultry manure alone resulted in a fruit yield of 50.23 t ha^−1^. The utilization of FYM resulted in the lowest fruit yield, with a modest 40.18 t ha^−1^. Contrary to the findings of Tao et al. [55], who reported no significant positive effect on fruit quantity when utilizing chemical fertilizers alongside PM for cherry tomatoes, our results yielded contrasting outcomes, which could potentially be attributed to methodological differences (e.g., variations in the types and timing of application of chemical fertilizers or the specific cultivars used), variability in plant response, environmental factors, or chance variation. In line with the findings of Basnet et al. [58] and Prashanti et al. [79], our results also indicated that the combination of 50 % NPK + 50 % poultry manure resulted in the highest yield increase, followed by a statistically similar treatment of 50 % NPK + 50 % farmyard manure for radish and cucumber cultivation, respectively.

**Table 7:** Fruit yield influenced by different organic and inorganic fertilizers.

Nutrient sources	Fruit yield (t ha^−1^)
Recommended dose of NPK	42.40^b^
100 % poultry manure	50.23^a^
100 % farmyard manure	40.18^b^
50 % NPK + 50 % poultry manure	55.56^a^
50 % NPK + 50 % farmyard manure	51.55^a^
Grand Mean	47.98
SEm (±)	1.86
LSD (0.05)	5.75
F-test	***
CV%	7.78

Data in columns with the same letters in DMRT are not significantly different (p = 0.05); SEm (±) = standard error of the mean; CV = coefficient of variation; LSD = least significant difference; *** = significant at p < 0.001.

The ideal method is to halve the application of organic manure and combine it with an appropriate quantity of inorganic fertilizer to achieve a balanced nutrient supply [79]. The utilization of mixed fertilizers provides bitter gourds with enhanced nutrient availability compared with the use of individual fertilizers [79]. PM exhibits two significant properties: its capacity for gradual nutrient release and its enduring impact on crops [28]. These characteristics contribute to the gradual availability of nutrients in the soil, fostering the build-up of soil humus and nutrient reserves. Consequently, the uptake and translocation of nutrients within the plant's vascular system are enhanced, especially during periods of water scarcity or drought stress [28]. The synergistic utilization of organic and chemical fertilizers has a positive effect on biological yield [37]. The application of these fertilizers influences moisture and nutrient uptake, retention, and availability, ultimately resulting in higher biological yield [37,80]. Research findings indicate that integrated fertilizer treatments, particularly the use of poultry manure, have shown effective suppression of 2,2-diphenyl-1-picrylhydrazyl (DPPH) radical, displaying the highest DPPH antioxidant activity [28]. Kumar et al. [81] emphasize that free radicals, such as superoxide, can cause oxidative damage to various metabolic pathways and disrupt the physiological functions of plants. However, the presence of antioxidant compounds in fertilizers acts as a free-radical scavenger, neutralizing these harmful free radicals [82]. Fertilizers play a noteworthy role in meeting the N requirements of photosynthetic pigments and plant cell proteins, as chlorophyll and carotenoids are inherently associated with proteins [57]. By supplying the necessary nitrogen, fertilizers contribute to an increase in the quantity of these pigments in crops. This increase in chlorophyll and carotenoid contents subsequently leads to improved crop productivity [28,83,84]. The combination of PM and NPK fertilizer application plays a role in soil moisture retention and improves nutrient availability for plants, leading to increased assimilate synthesis. Consequently, these fertilizers contribute to enhanced photosynthate synthesis, resulting in increased plant yield [80]. Moreover, the improvement in yield through integrated nutritional approaches can be ascribed to elevated levels of microbial and enzymatic activities, which positively influence nutrient cycling and availability [85]. The utilization of INM, which continuously supplies plant nutrients throughout the growth period and critical stages, in combination with NPK contributes to higher fruit yield, improved nutrient uptake, and increased plant vigor. Additionally, this combination enhances Mg, nitrogen, and Zn uptake by elevating soil pH. Therefore, the INM treatment increased fruit production [57].

These findings also suggest that fruit yield does not show significant improvement beyond a certain threshold when excessive amounts of inorganic fertilizer (in the case of the recommended dose of NPK) are applied [86,87]. This excessive application negatively affected fruit yield. The sole application of inorganic fertilizer has been shown to be excessive and can impede the growth and accumulation of biomass [26]. Moreover, it also leads to soil acidification and hardening, further emphasizing the adverse effects of excessive sole application [55].

### 3.3 Economics of bitter gourd production

Table 8 provides valuable insights into the economic viability of different nutrient sources in bitter gourd production, aiding decision-making by farmers and industry stakeholders. The 50 % NPK +50 % poultry manure treatment resulted in the lowest treatment cost of $394.96, resulting in a total production cost of $2524. This approach yields a substantial gross return of $10468, leading to a robust net return of $7944 and the highest benefit-cost ratio of 3.14, followed by 50 % NPK +50 % farmyard manure with a treatment cost of $635.90, leading to a total production cost of $2768. This combination results in a gross return of $9712, leading to a net return of $6944 and the second-highest benefit-cost ratio of 2.50. The 100 % poultry manure treatment, with a treatment cost of $704.34 and gross return of $9464, yielded a favorable net return of $6630 and a benefit-cost ratio of 2.33. The recommended dose of NPK treatment incurs a cost of $972.19, resulting in a total production cost of $3107. This investment yields a gross return of $7988, leading to a net return of $4881, with a benefit-cost ratio of 1.57. In contrast, 100 % farmyard manure treatment had the highest treatment cost of $1185.85, leading to the highest total production cost of $3322. The gross return is the lowest ($7571) among the treatments, resulting in a net return of $4249 with the lowest benefit-cost ratio of 1.27.

**Table 8:** Economics of bitter gourd production using organic and inorganic fertilizers.

Nutrient sources	Treatment cost (US$ ha^−1^)	Total cost of production (US$ ha^−1^)	Gross return (US$ ha^−1^)	Net return (US$ ha^−1^)	Benefit cost ratio
Recommended dose of NPK	972.19	3107^d^	7988^c^	4881^c^	1.57^d^
100 % poultry manure	704.34	2834^c^	9464^b^	6630^b^	2.33^c^
100 % farmyard manure	1185.85	3322^e^	7571^d^	4249^d^	1.27^e^
50 % NPK + 50 % poultry manure	394.96	2524^a^	10468^a^	7944^a^	3.14^a^
50 % NPK + 50 % farmyard manure	635.90	2768^b^	9712^b^	6944^b^	2.50^b^
Grand Mean	778.64	2911.10	9041	6130	2.16
SEm (±)	–	2.74	150.2	150.5	0.05
LSD (0.05)	–	5.97	327.2	328	0.11
F-test	–	***	***	***	***
CV%	–	0.1	2.3	3.5	3.5

Data in columns with the same letters in DMRT are not significantly different (p = 0.05); SEm (±) = standard error of the mean; CV = coefficient of variation; LSD = least significant difference; *** = significant at p < 0.001.

Integrated nutrient management has yielded a notably higher benefit-cost ratio (B:C ratio), which is primarily attributed to two key factors. First, it is attributable to reduced treatment costs, and second, it is linked to increased crop yields. The amalgamation of both synthetic and organic fertilizers facilitates the provision of a well-balanced and synergistic nutrient supply to crops, effectively optimizing their growth and bolstering their yield potential. Consequently, this approach often leads to elevated crop productivity compared with the utilization of singular fertilizer types [57]. As shown in Table 7, integrated fertilizer treatments consistently resulted in higher yields, resulting in enhanced returns [88]. Additionally, integrated combinations of fertilizers are often more economically efficient and readily accessible than pure NPK fertilizers or exclusive organic alternatives. This not only reduces the overall treatment costs but also ensures the delivery of essential nutrients crucial for crop development [57,88,89]. These results contrast with those of Prasad et al. [88] in bottle gourd, where the recommended NPK dose resulted in the highest gross and net return and benefit-cost (B:C) ratio, followed by integrated nutrient management, as their yield outcomes were statistically similar to those achieved in integrated fertilizer treatments. However, in our study, the yield was relatively higher in the integrated fertilizer treatments, which consequently led to the highest gross and net returns and B:C ratio than recommended chemical fertilizer treatment.

### 3.4 Pearson correlation analysis among various growth and yield parameters

**Fig. 1:**
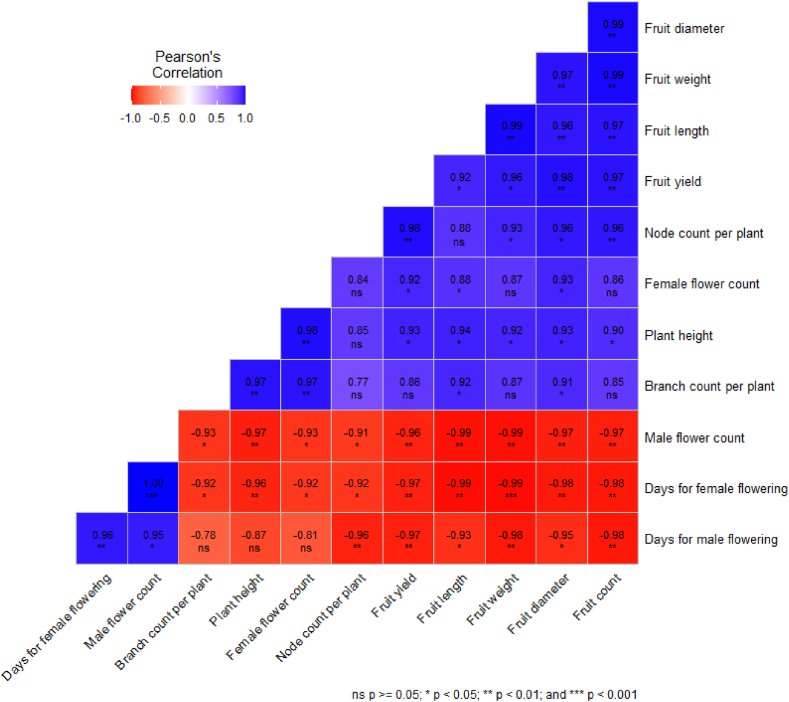
Correlation among various parameters considered.

Fig. 1 illustrates the correlation coefficients, which depict the relationships between the various pairs of variable parameters examined in this study. Each value in the table represents the correlation coefficient between the two variables. Plant height has a strong positive correlation with branch count plant^−1^ (0.97) and node count plant^−1^ (0.85), indicating that taller plants tend to have more branches and nodes [90]. The days to first male flowering and days to first female flowering were strongly negatively correlated with plant height, branch count, and node count, indicating that they tended to have shorter and fewer branches and fewer nodes. Plants with higher male and female flower counts demonstrated a strong negative correlation between days to male flowering and days to female flowering, suggesting that increased flower counts are associated with earlier male and female flowering. Fruit length, fruit diameter, fruit weight, fruit number, and fruit yield were strongly positively correlated with each other, which is consistent with the results of Omondi et al. [91] for baobab fruit. This implies that larger fruits (in terms of length, diameter, weight, and number) tend to have higher yields. Fruit length, diameter, weight, and number also had strong negative correlations with days to male and female flowering, suggesting that plants that take longer to flower tend to produce smaller and fewer fruits. The correlation coefficients provide an assessment of the magnitude and direction of the linear relationship between variables. However, it is important to note that the correlation does not imply causation, and other unaccounted factors in the experiment may also influence the observed relationships.

Various factors, including cultivar, agronomic factors (e.g., irrigation, planting methods, and spacing), and climatic factors (e.g., temperature, relative humidity, growing season, rainfall, and soil fertility) could have influenced the outcomes of the production process, potentially leading to different results [57]. Despite these positive results, it is important to acknowledge that the findings may not be directly generalizable to other cultivation areas due to potential variations in soil characteristics and other influential factors.

### 3.5 Policy implication of the study

#### 3.5.1 Promotion of integrated fertilizer management

The findings of this study emphasize the effectiveness of integrated organic and inorganic fertilizers, specifically a blend of 50 % NPK and 50 % poultry manure, compared to chemical and organic fertilizers used alone in bitter gourd cultivation to enhance the growth and yield of bitter gourd. Policymakers and agricultural authorities should consider promoting and disseminating knowledge regarding sustainable practices to smallholder farmers. Training programs and extension services can be organized to educate farmers about the benefits of combining organic and inorganic nutrient sources with a focus on cost-effectiveness and sustainability [92]. Ensure that farmers have access to the training, materials, and resources needed to effectively adopt these practices. This could involve providing subsidies or technical support to farmers who opt for INM, thus reducing the environmental impact of chemical fertilizers [93].

#### 3.5.2 Access to organic nutrient sources

Research demonstrates that organic nutrient sources, such as PM and FYM, can significantly contribute to crop growth and quality. Policymakers should explore ways to make organic resources more accessible to farmers. Initiatives such as subsidizing organic fertilizer costs, especially given the relatively higher price of organic manure as presented in Table 8, promoting community-based composting, and facilitating the collection and distribution of organic manures, can contribute to improving access to and the adoption of these sustainable practices.

#### 3.5.3 Research and extension support

Policymakers should allocate resources for additional research on larger scales and diverse ecological regions to further validate and extend the positive findings of this study. Allocate funding for further research on bitter gourd cultivation practices. This includes investigating different varieties, pest control methods, and irrigation strategies that can complement the use of integrated fertilizers to maximize crop yield and quality. Collaborative efforts between research institutions, government agencies, and agricultural organizations can facilitate the development of region-specific fertilizer-management guidelines. These guidelines should be continually assessed and adapted to changing conditions to ensure long-term sustainability and resilience in bitter gourd cultivation. Moreover, extension services should be strengthened to ensure that farmers receive timely guidance on implementing integrated nutrient-management strategies [94].

#### 3.5.4 Sustainable agriculture promotion

This study highlights the potential of integrated fertilization practices to enhance yield and promote sustainable and environmentally friendly agricultural practices. Policymakers can integrate these findings into broader agricultural sustainability initiatives aimed at reducing the agricultural environmental footprint [57]. These initiatives should prioritize minimizing nutrient runoff and soil degradation, while emphasizing the importance of reducing chemical fertilizer dependence and adopting eco-friendly approaches. Encouraging practices that simultaneously reduce soil and water pollution while enhancing agricultural productivity is crucial for a more sustainable future [57].

## 4 Conclusion and recommendations

Bitter gourds showed a significant response to several organic and inorganic nutrient sources on growth metrics. The use of integrated organic and inorganic fertilizers enhances yield and yield-attributing traits, establishing them as viable nutrient sources for bitter gourd cultivation. The application of a 50 % NPK and 50 % poultry manure blend significantly improved multiple growth parameters, including plant height, branch count plant^−1^, node count plant^−1^, male and female flower counts, fruit length, fruit diameter, individual fruit weight, fruit count, fruit yield, net return, and the highest benefit cost ratio. Moreover, this treatment reduced the time to the first flowering. These results indicate that a combination of 50 % NPK and 50 % poultry manure can serve as a highly effective nutrient source for promoting growth and maximizing bitter gourd yield. Notably, this integrated approach was statistically comparable to the combination of 50 % NPK and 50 % farmyard manure. Additionally, chemical fertilizers did not demonstrate superiority in any of the evaluated traits. Based on the observed outcomes, the use of integrated fertilizers, particularly 50 % NPK +50 % poultry manure, is recommended as a superior alternative to chemical and organic fertilizers alone. This approach not only enhances yield but also promotes sustainable and environmentally friendly agricultural practices. Despite these positive results, it is important to note that further research on larger scales and in different ecological regions is warranted to validate these findings and to promote sustainable bitter gourd production. Bitter gourd cultivation can be influenced by factors such as cultivar, agronomic practices, and climatic conditions; therefore, it is essential to confirm the efficacy of this fertilizer management strategy across a broader spectrum of contexts.

## Funding statement

This research did not receive any specific grant from funding agencies in the public, commercial, or not-for-profit sectors.

## Data availability statement

Data will be made available on request.

## CRediT authorship contribution statement

**Sudip Ghimire:** Writing – review & editing, Writing – original draft, Visualization, Validation, Supervision, Software, Resources, Project administration, Methodology, Investigation, Funding acquisition, Formal analysis, Data curation, Conceptualization. **Bidhya Poudel Chhetri:** Writing – review & editing, Writing – original draft, Visualization, Validation, Supervision, Software, Resources, Project administration, Methodology, Investigation, Funding acquisition, Formal analysis, Data curation, Conceptualization. **Jiban Shrestha:** Writing – review & editing, Supervision, Visualization.

## Declaration of competing interest

The authors declare that they have no known competing financial interests or personal relationships that could have appeared to influence the work reported in this paper.
